# Evaluation of degree centrality and neurological outcomes in patients with herpes simplex encephalitis

**DOI:** 10.3389/fneur.2025.1588294

**Published:** 2025-09-03

**Authors:** Dongzhi Liu, Xiaoyu Liu, Dong Zhu, Yuanfei Chen, Jueyue Yan, Jingchen Zhang, Hongqin He

**Affiliations:** ^1^Department of Emergency Medicine, Shaoxing Central Hospital, Shaoxing, China; ^2^Infectious Disease Department, The Second People’s Hospital of Quzhou, Quzhou, China; ^3^Department of Critical Care Medicine, The First Affiliated Hospital, Zhejiang University School of Medicine, Hangzhou, China; ^4^Radiology Department, Shaoxing Central Hospital, Shaoxing, Zhejiang, China

**Keywords:** herpes simplex encephalitis, degree centrality, resting-state fMRI, neurological outcome, functional brain connectivity

## Abstract

**Objective:**

This study aimed to investigate the association between changes in cerebral degree centrality (DC) and clinical outcomes in patients with herpes simplex encephalitis (HSE).

**Methods:**

All participants underwent functional magnetic resonance imaging (fMRI), and DC analysis was used to identify voxels that showed changes in whole-brain functional connectivity with other voxels. DC was evaluated using the fMRI graph method, and comparisons between HSE patients and controls were performed. The National Institutes of Health Stroke Scale (NIHSS), the Glasgow Coma Scale (GCS), and the modified Rankin Scale (mRS) were assessed for all HSE patients.

**Results:**

A total of 70 HSE patients and 76 controls were included in our data analysis. In the HSE group, DC values were increased in the right thalamus, the left lingual gyrus, and the right hippocampus, while they were decreased in the left insula (all *p* < 0.001). In our HSE cohort, the NIHSS score was significantly associated with DC changes in the right thalamus (*r* = −0.282, *p* = 0.018), the left insula (*r* = −0.301, *p* = 0.011), the left lingual gyrus (*r* = −0.270, *p* = 0.024), and the right hippocampus (*r* = 0.238, *p* = 0.047). We also found that DC changes in the right thalamus (*r* = 0.241, *p* = 0.044), the left insula (*r* = 0.254, *p* = 0.034), and the left lingual gyrus (*r* = 0.275, *p* = 0.021) were significantly associated with higher GCS scores. After 90 days, neurological deficit was assessed via the mRS; we found that mRS was associated with the right thalamus (*r* = −0.272, *p* = 0.023) and the left lingual gyrus (*r* = −0.270, *p* = 0.024).

**Conclusion:**

Cerebral intrinsic connectivity changes as assessed by DC in HSE were associated with neurological deficits (as assessed by the NIHSS score), level of consciousness (as assessed by the GCS score), and functional disability. Our findings provide novel insights into the neural mechanisms underlying HSE-related neurological deficits and inform the development of targeted therapeutic interventions.

## Introduction

Herpes simplex encephalitis (HSE), a rare but severe central nervous system (CNS) infection caused by the herpes simplex virus, is associated with significant neurological morbidity and mortality (1, 2). Despite advancements in early diagnosis and antiviral therapies, many HSE survivors experience long-term neurological deficits, including cognitive dysfunction, motor impairments, and altered consciousness (2). Understanding the neurophysiological mechanisms underlying these deficits is crucial for improving diagnosis, treatment, and rehabilitation strategies.

Resting-state functional magnetic resonance imaging (rs-fMRI) has emerged as a powerful tool for investigating brain network connectivity in various neurological conditions. Among the metrics derived from rs-fMRI, degree centrality (DC)—which measures the importance of a brain region within the global functional network—has gained attention for its ability to quantify alterations in cerebral network organization (3). DC reflects the number of direct connections a given brain region has with other regions, providing insights into the brain’s functional integration and segregation (3). Aberrant DC has been linked to cognitive and neurological impairments in a variety of brain conditions (4, 5); however, its role in HSE remains poorly understood.

This study aims to assess cerebral degree centrality in patients with HSE compared to healthy controls, with a particular focus on identifying brain regions that exhibit significant connectivity alterations. Furthermore, we aim to explore the relationship between changes in cerebral degree centrality and clinical measures of neurological function, including the modified Rankin Scale (mRS), the National Institutes of Health Stroke Scale (NIHSS), and the Glasgow Coma Scale (GCS). By examining these associations, we seek to elucidate the potential role of network connectivity disruptions in the pathophysiology of HSE and their contribution to clinical outcomes. Understanding these relationships could provide novel insights into the neural mechanisms underlying HSE-related neurological deficits and inform the development of targeted therapeutic interventions.

## Methods

This single-center cohort study was performed at the First Affiliated Hospital, Zhejiang University School of Medicine, China, between November 2023 and November 2024. We assessed all medical information of patients who were clinically diagnosed with encephalitis (International Classification of Diseases, Ninth Revision code 054.3) and exhibited cerebrospinal fluid (CSF) positivity for herpes simplex virus (HSV) DNA in polymerase chain reaction analysis. All patients underwent neuroimaging. The ethics committee of the First Affiliated Hospital, Zhejiang University School of Medicine, approved the study, which was conducted in accordance with the Declaration of Helsinki (IIT20230253B-R2). Additionally, the study also followed the Strengthening the Reporting of Observational Studies in Epidemiology (STROBE) reporting guidelines.

### HSE patients

Our inclusion criteria were as follows: admission to the intensive care unit (ICU) or the neurology department with a hospital stay of 24 h or more, a clinical suspicion of acute encephalitis, and a CSF polymerase chain reaction test positive for HSV DNA during hospitalization. We diagnosed possible acute encephalitis according to international guidelines (6), which require acute changes in mental status or behavior lasting 24 h or longer, accompanied by at least two of the following manifestations: (1) fever within 72 h before or after presentation, (2) generalized or partial seizures, (3) new-onset focal neurological findings, (4) lumbar puncture with a CSF white blood cell count ≥ 5 cells/uL, or (5) neuroimaging or electroencephalogram aberrations suggestive of encephalitis. The exclusion criteria were as follows: neuroimaging not performed or performed 14 days after ICU admission, preexisting neurological disease that could interfere with cerebral MRI analysis (such as a brain tumor or severe traumatic brain injury), poor-quality MRI, or incomplete clinical information.

#### Clinical information

Patient’s histories, clinical, laboratory, and neuroimaging data were gathered from medical records. The neuropsychological examination was assessed at ICU admission. Each participant underwent a neuropsychological assessment. Neurological deficits were assessed using the National Institutes of Health Stroke Scale (NIHSS) at admission (7). The global cognitive level was assessed using the Mini-Mental State Examination (MMSE) and the Montreal Cognitive Assessment (MoCA). With a total score of 30, both the MoCA and MMSE are brief neuropsychological screening tools; a higher score indicates better cognition (8, 9). Mental status at ICU admission was graded using the Glasgow Coma Scale (GCS, range, 3–15), with coma defined as a GCS score of less than 8 (10).

Controls for our study were enrolled through advertisements for residents who attended our hospital for health examinations. Individuals who denied a history of neurologic conditions when they visited our hospital for routine examinations and those who did not show any abnormality on MRI were enrolled in our study as controls. Medical histories were recorded for all individuals. Lifestyle, vascular risk factors, and clinical information were recorded for all controls.

#### Outcome assessment

Using the Modified Rankin Scale (mRS) (11, 12), the functional outcome was graded at 90 days after ICU admission. The mRS assesses the degree of disability or dependence in daily activities in brain-injured patients on a scale of 0 to 6, with a lower score denoting fewer disabilities or less dependence. A systematic assessment was conducted based on available follow-up consultation records and/or information provided by doctors. A poor functional outcome at 90 days was defined by a score on the mRS of 3–6 (indicating moderate to severe disability or death). A good functional outcome at 90 days was defined by a score on the mRS of 0–2 (indicating slight disability or no symptoms at all). Patients who were discharged home with functional independence before 90 days were considered to have a good functional outcome.

#### Neuroimaging examination

An MRI examination was performed within the first week after ICU admission, and patients were considered for the examination. A Siemens 3-Tesla Trio Tim MRI scanner (Siemens AG, Munich, Germany) equipped with a 32-channel head coil was used to image the brain of each participant. Functional images were obtained using an echo-planar imaging sequence with the following parameters: 33 axial slices, with thickness/gap = 4.8/0 mm, an in-plane resolution = 64 × 64, a repetition time (TR) = 2,000 ms, an echo time (TE) = 30 ms, a flip angle = 90 degrees and a field of view (FOV) = 200 × 200 mm^2^. Each condition consisted of 200 functional volumes. For registration purposes, high-resolution anatomical images were acquired using a 3D magnetization-prepared rapid gradient echo T1-weighted sequence (T1/TR/TE = 900/1,900/2.48 ms, flip angle = 9 degrees, 128 slices, FOV = 256 × 256 mm^2^, 1 × 1 × 1 mm^2^ resolution) of each subject.

### Data processing

All rsfMRI data preprocessing was performed using SPM8 (http://www.fil.ion.ucl.ac.uk/spm) and the Data Processing Assistant for Resting-State fMRI software (REST; http://www.restfmri.net). The preprocessing steps included slice-timing correction, head motion correction, linear trend removal, spatial smoothing with a 6-mm Gaussian kernel in all three directions, and spatial normalization to Montreal Neurological Institute (MNI) space with a resampling resolution of 3 × 3 × 3 mm^3^. All smoothed images were then filtered using a typical temporal bandpass filter (0.01–0.08 Hz) to reduce low-frequency drift and high-frequency physiological noise from respiratory and cardiac activities. Linear trends were also removed from each time series.

### Calculation of DC

Voxel-based whole-brain correlation analysis on the pre-processed fMRI data was performed to calculate voxel-wise DC, as previously described (13). Pearson’s correlation coefficients (r) were calculated between all pairs of brain voxels within the gray matter mask. We then converted the Pearson’s correlation data to normally distributed Fisher’s Z-scores, and constructed the whole-brain functional network by thresholding each correlation at *r* > 0.25 as previously reported (14). The DC value for a given voxel was calculated as the sum of its significant connections at the individual level. Voxel-wise DC values were also converted into a Z-score map using the Fisher-Z transformation to improve normality. Positive correlations were considered in the DC calculation due to the uncertainty of interpretation and detrimental effects on test–retest reliability.

To assess the difference in DC values between HSE patients and controls, a two-sample *t*-test was performed using REST. AlphaSim, a program based on Monte Carlo simulations and implemented in Analysis of Functional NeuroImages (AFNI), was used for multiple comparison corrections. Monte Carlo simulations determine the random distribution of cluster size for a given per-voxel threshold. Statistical significance was defined as a *p*-value of < 0.05 and a cluster size of > 198 voxels, corresponding to a corrected *p*-value of < 0.05. The correction was confined within the gray matter mask and was determined using Monte Carlo simulations.

#### Statistical analysis

Continuous variables were displayed as mean ± standard deviation, and categorical variables as number (%) as appropriate. To assess the correlation between DC changes and clinical features of HSE, partial correlation was used while adjusting for risk factors (age, gender, hypertension, and dyslipidemia). *p*-values of less than 0.05 were considered statistically significant. R statistical software (version 4.4.3) was used for all statistical analyses.

## Results

The characteristics of the study cohort are shown in Table 1. Overall, 70 HSE patients and 76 controls were included in our data analysis. Of the 70 HSE patients, 29 (41.43%) had a history of hypertension, and 36 (51.43%) had epileptic seizures. The median NIHSS score was 19 [13, 22], while the median GCS score was 10 (6, 12) in the HSE cohort. Of note, the median temperature was 38.9 °C, and 61 patients had a fever.

**Table 1:** Characteristics of study participants.

Variable	Controls *N* = 76)	HSE *N* = 70)	*P*-value
Age, years	30.92 ± 6.90	32.14 ± 7.07	0.293
Male patients, *n* (%)	63 (82.89)	57 (81.43)	0.988
Hypertension, *n* (%)	25 (32.89)	29 (41.43)	0.371
Temperature, °C		38.9 [38.5, 39.9]	
Fever (>38.3 °C)		61 (87.14)	
Epileptic seizures		36 (51.43)	
NIHSS		19 [13, 22]	
CSF pressure, mmHg		30.92 ± 6.90	
CSF protein		30.92 ± 6.90	
Follow-up			
GCS		10 [6, 12]	
mRS		2 [1, 3]	

NIHSS, National Institutes of Health Stroke Scale; GCS, Glasgow Coma Scale; mRS, modified Rankin Scale; CSF, cerebrospinal fluid.

**Figure 1:**
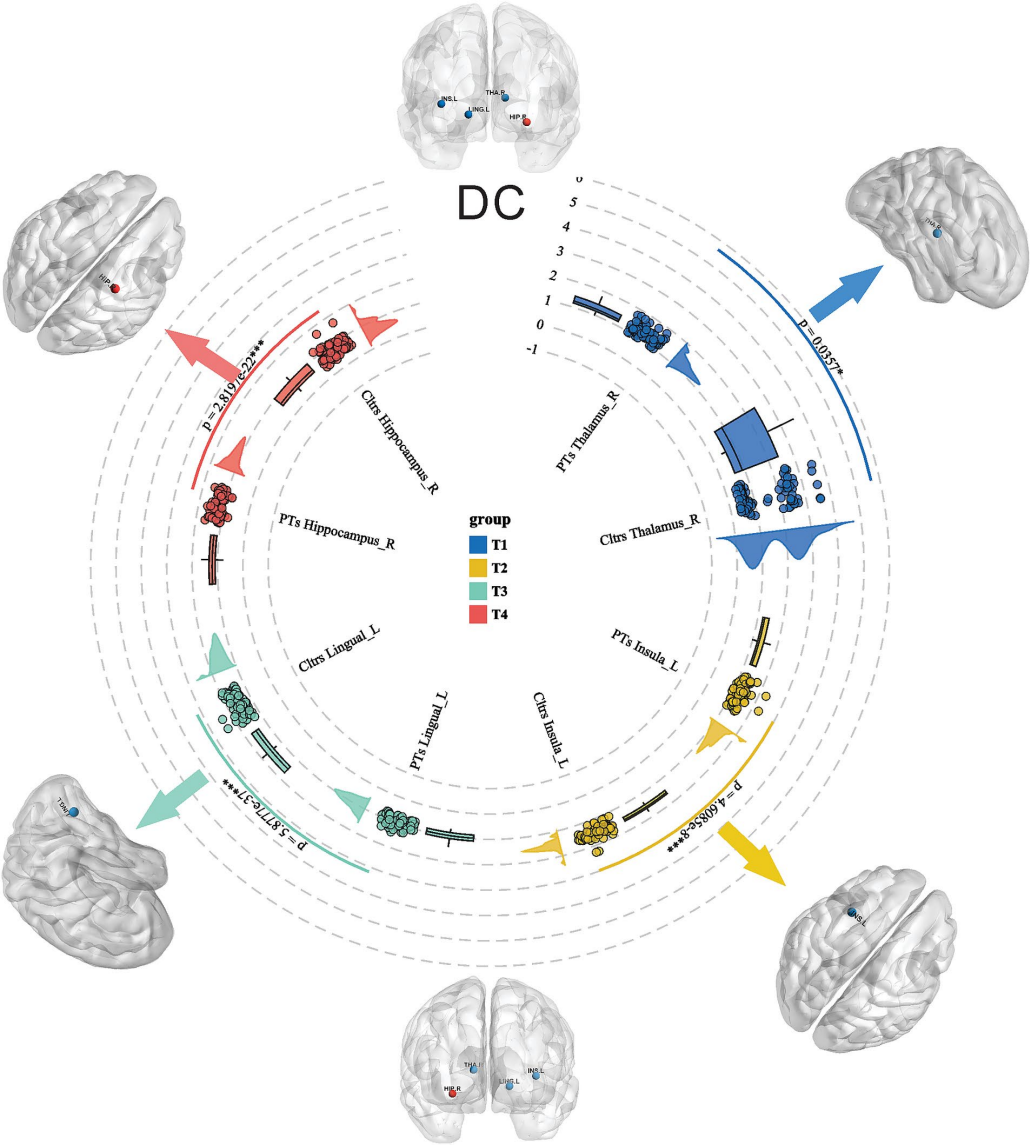
Comparison of DC values in HSE and controls. HSE showed increased DC values in the right thalamus, the left lingual gyrus, and the right hippocampus, while the DC value was decreased in the left insula.

A total of 90 brain regions involved in Anatomical Automatic Labeling (AAL) were analyzed in the study. AAL partitions are provided by the Montreal Neurological Institute (MNI). There are 116 regions in the AAL template, but only 90 belong to the brain. After correction for multiple comparisons, HSE patients showed increased DC values in the right thalamus, the left lingual gyrus, and the right hippocampus, while the DC value was decreased in the left insula (all *p* < 0.001; Figure 1). Routine structural MRI revealed lesions predominantly involving the medial temporal lobes (81.4%), the insular cortex (70.0%), and the basal frontal areas (54.3%) in HSE patients, which was consistent with the characteristic limbic system involvement of HSE (Supplementary Table S1). Representative MRI findings are illustrated in Supplementary Figure S1.

### DC findings and clinical outcomes

In our HSE cohort, the NIHSS score was significantly associated with DC changes in the right thalamus (*r* = −0.282, *p* = 0.018), the left insula (*r* = −0.301, *p* = 0.011), the left lingual gyrus (*r* = −0.270, *p* = 0.024), and the right hippocampus (*r* = 0.238, *p* = 0.047). We also found that DC changes in the right thalamus (*r* = 0.241, *p* = 0.044), the left insula (*r* = 0.254, *p* = 0.034), and the left lingual gyrus (*r* = 0.275, *p* = 0.021) were significantly associated with GCS.

**Figure 2:**
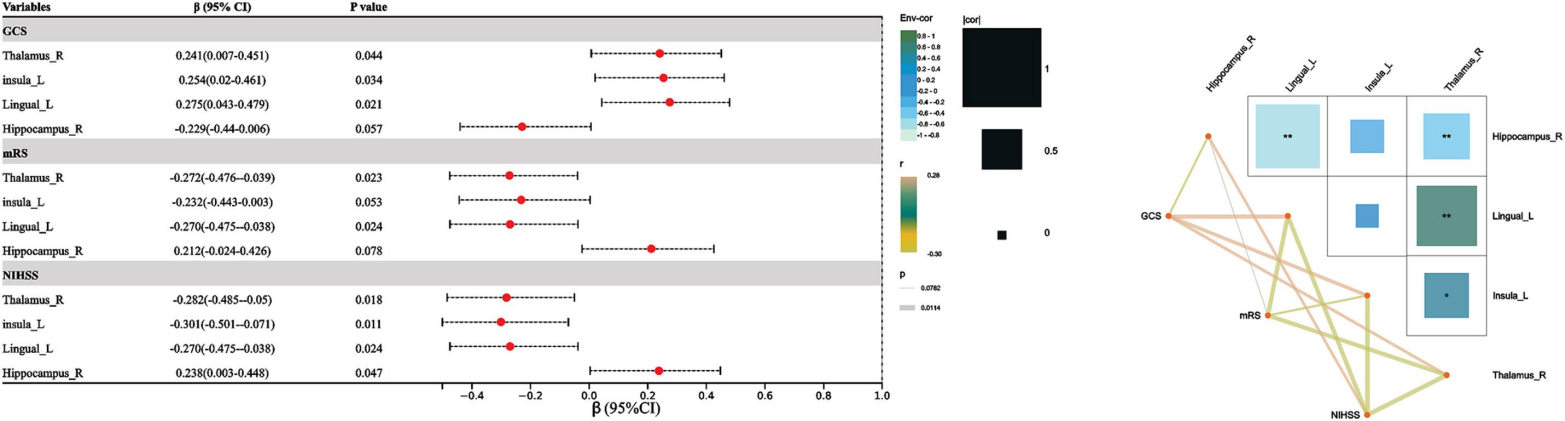
Association between DC values and clinical outcomes in HSE. The NIHSS was significantly associated with DC changes in the right thalamus, the left insula, the left lingual gyrus, and the right hippocampus. It was also found that DC changes in the right thalamus, the left insula, and the left lingual gyrus were significantly associated with the GCS.

After 90 days, neurological deficit was assessed via the mRS; we found that a higher mRS score was associated with the right thalamus (*r* = −0.272, *p* = 0.023) and the left lingual gyrus (*r* = −0.270, *p* = 0.024). No significant association was found between the mRS and the left insula (*r* = −0.232, *p* = 0.053) or the right hippocampus (*r* = 0.212, *p* = 0.078). Figure 2 shows the association between DC values and clinical outcomes in HSE.

## Discussion

In this single-center study, we evaluated the intrinsic dysconnectivity pattern of brain functional networks in HSE using DC analysis. We showed that HSE had increased DC values in the right thalamus, the left lingual gyrus, and the right hippocampus, while they were decreased in the left insula. We also showed that intrinsic connectivity in the brains of patients with HSE was associated with NIHSS, GCS, and mRS scores.

In patients with herpes simplex encephalitis (HSE), degree centrality (DC) analysis revealed distinct alterations in functional brain network connectivity compared to controls. Specifically, DC values were significantly increased in the right thalamus, the left lingual gyrus, and the right hippocampus, suggesting heightened functional connectivity or compensatory hyperactivation in these regions, which are critical for sensory integration (15), visual processing (16), and memory consolidation (17), respectively. Conversely, DC values were decreased in the left insula, a region involved in emotional regulation and interoceptive awareness (18, 19), indicating impaired network integration or functional disconnection. These findings highlight a pattern of disrupted network dynamics in HSE, where compensatory mechanisms in key cognitive and sensory regions may coexist with functional deficits in areas governing higher-order socioemotional processing, reflecting the complex interplay of neuroinflammation and neurodegeneration in this condition.

The National Institutes of Health Stroke Scale (NIHSS) assesses the degree of neurological impairment, and its significant association with changes in right thalamus, left insula, left lingual gyrus, and right hippocampal DC (degree centrality) may be related to the functional roles of these brain regions and the pathological features of herpes simplex virus encephalitis (HSE). As a key node in sensorimotor information integration, the decreased DC of the right thalamus may reflect the disconnection of the thalamic-cortical network, leading to sensorimotor dysfunction (such as the decreased motor score in the NIHSS). The left insula is involved in autonomic nervous system regulation and consciousness maintenance (18). Abnormal DC may exacerbate language disorders (such as aphasia) and fluctuations in consciousness levels, directly affecting the NIHSS score. The left lingual gyrus is involved in visual information processing, and changes in its DC may correspond to visual field defects or visual spatial neglect (NIHSS vision item). Abnormal DC in the right hippocampus may indicate impaired memory coding function, which is related to the orientation score in the NIHSS. HSE inflammation involves the limbic system and temporal lobe, further aggravating network separation in these regions and thereby strengthening the association between the NIHSS score and DC.

The Glasgow Coma Scale (GCS), which assesses the level of consciousness, was significantly associated with changes in the DC of the right thalamus, the left insula, and the left lingual gyrus due to the central role of these regions in maintaining arousal and perceptual integration. The right thalamus is an important hub of the ascending reticular activation system (20), and the decrease of DC may lead to the obstruction of thalamic-cortical excitatory transmission, and directly inhibit the arousal state (GCS score decline). The left insula regulates consciousness by integrating visceral sensory and emotional signals (21). Abnormal DC may disrupt the autonomic basis of consciousness maintenance. DC changes in the left lingual gyrus may affect the input of visual information to the higher cortex, resulting in decreased environmental perception, which in turn reduces the GCS score. However, the hippocampus is mainly involved in memory rather than in the immediate regulation of consciousness, so there is no significant correlation between the change of DC and the GCS score. The focal inflammation in HSE is prone to damaging these key network nodes, exacerbating the disturbance of consciousness through the interruption of functional connectivity and forming the pathological association between DC and GCS.

The modified Rankin Scale (mRS) assesses functional disability, and its association with DC changes in the right thalamus and the left lingual gyrus in herpes simplex encephalitis (HSE) reflects the critical roles these regions play in motor, sensory, and cognitive integration. The right thalamus, as a central relay station for sensory and motor signals, is crucial for coordinated movement and sensory processing. DC changes in this region may indicate disrupted thalamo-cortical connectivity, leading to motor and sensory impairments that directly contribute to higher mRS scores (greater disability). Similarly, the left lingual gyrus, involved in visual processing and visuospatial integration, is essential for daily activities requiring visual perception. DC abnormalities in this region may result in visual deficits or impaired visuospatial awareness, further contributing to functional disability. In HSE, inflammation and network disruption in these areas likely exacerbate connectivity impairments, linking DC changes in functional outcomes measured using the mRS.

The main strengths of our study are the inclusion of a relatively large cohort compared with previous studies, the standardized assessment of functional outcome, and the evaluation of the connectivity changes in the brain using DC. To our knowledge, this is the first study to explore the connectivity changes in HSE patients with definite outcomes.

However, our results must be interpreted with caution, and a number of limitations should be kept in mind. The first is the observational, cross-sectional design of the study, which limits the interpretation of the results with respect to cause and effect. The results were obtained from a small town in Zhejiang province of China, which limits the generalizability of the data to the general population of China. Moreover, patients with incomplete clinical information were excluded, which might introduce selection bias. Another limitation of our study was the timing of the MR imaging, which was conducted during the acute phase (within a week) of the disease. Furthermore, investigating the dynamic changes from the acute to chronic phases may provide more comprehensive insights.

In this study, we showed that intrinsic connectivity changes, as assessed using DC in HSE patients, were associated with neurological deficits (as assessed by the NIHSS), level of consciousness (as assessed by the GCS), and functional disability. Our findings provide novel insights into the neural mechanisms underlying HSE-related neurological deficits and inform the development of targeted therapeutic interventions.

## Data Availability

The data analyzed in this study is subject to the following licenses/restrictions: The dataset used in this study is owned by the First Affiliated Hospital of Zhejiang University School of Medicine. Access to the data is restricted to protect patient confidentiality and in compliance with institutional policies and applicable data protection regulations. The data may only be shared with authorized researchers after a formal request and review process, ensuring that all data access and use adhere to ethical standards and legal requirements. Requests to access these datasets should be directed to Jueyue Yan, yan814122408@163.com.
