# Supplementary figures and images for: Evaluation of degree centrality and neurological outcomes in patients with herpes simplex encephalitis
Source: Front Neurol. 2025 Sep 3;16:1588294. doi: 10.3389/fneur.2025.1588294 (PMC12440766; doi:10.3389/fneur.2025.1588294)

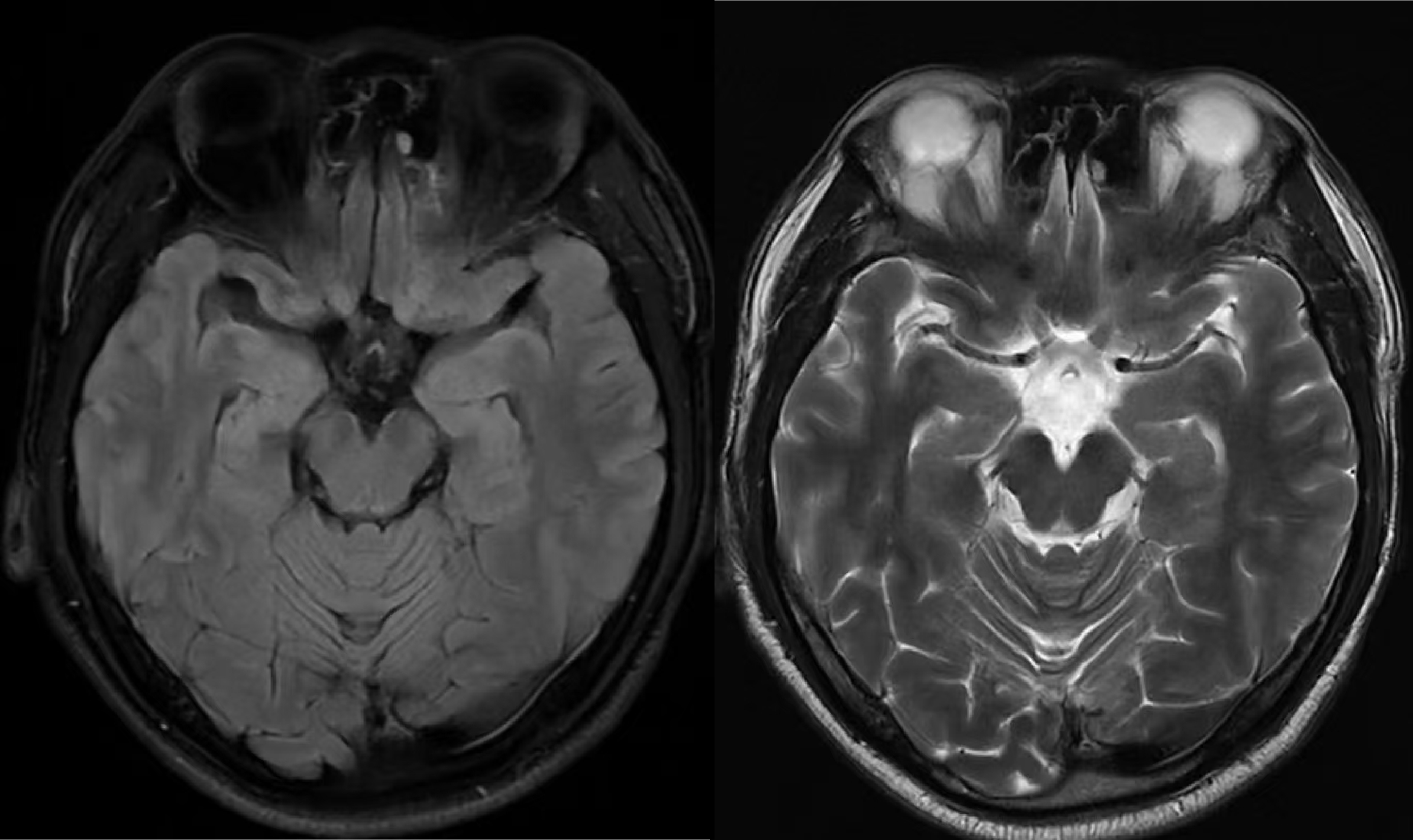

Supplement: Supplementary file 1 [file Image_1.tif]
