# Supplementary material for: Evaluation of degree centrality and neurological outcomes in patients with herpes simplex encephalitis
Source: Front Neurol. 2025 Sep 3;16:1588294. doi: 10.3389/fneur.2025.1588294 (PMC12440766; doi:10.3389/fneur.2025.1588294)
Supplement: Supplementary file 2 [file Table_1.docx]

# Table S1. Lesion Distribution on Routine Structural MRI in HSE Patients (N = 70)

| Brain Region Involved | No. of Patients (%) |
| --- | --- |
| Medial temporal lobe | 57 (81.4%) |
| Insular cortex | 49 (70.0%) |
| Basal frontal region | 38 (54.3%) |
| Thalamus | 22 (31.4%) |
| Occipital lobe | 11 (15.7%) |
| Bilateral involvement | 28 (40.0%) |
| No visible lesion on T1/FLAIR | 5 (7.1%) |
